# Rationale, design and initial results of an educational intervention to improve provider-initiated HIV testing in primary care

**DOI:** 10.1093/fampra/cmaa139

**Published:** 2020-12-26

**Authors:** Saskia J Bogers, Maarten F Schim van der Loeff, Nynke van Dijk, Karlijn Groen, Marije L Groot Bruinderink, Godelieve J de Bree, Peter Reiss, Suzanne E Geerlings, Jan E A M van Bergen

**Affiliations:** 1 Department of Internal Medicine, Division of Infectious Diseases, Amsterdam University Medical Centers, Location Academic Medical Center, University of Amsterdam, Amsterdam, the Netherlands; 2 Department of Infectious Diseases, Public Health Service of Amsterdam, Amsterdam, the Netherlands; 3 Department of General Practice, Amsterdam University Medical Centers, Location Academic Medical Center, University of Amsterdam, Amsterdam, the Netherlands; 4 Amsterdam Institute for Global Health and Development, Amsterdam, the Netherlands; 5 Department of Global Health, Amsterdam University Medical Centers, Location Academic Medical Center, University of Amsterdam, Amsterdam, the Netherlands; 6 HIV Monitoring Foundation, Amsterdam, the Netherlands; 7 STI AIDS Netherlands, Amsterdam, the Netherlands

**Keywords:** continuing medical education, general practice, HIV, primary care, sexually transmitted disease, quality improvement

## Abstract

**Objectives:**

In the Netherlands, general practitioners (GPs) perform two-thirds of sexually transmitted infection (STI) consultations and diagnose one-third of HIV infections. GPs are, therefore, a key group to target to improve provider-initiated HIV testing. We describe the design and implementation of an educational intervention to improve HIV testing by Amsterdam GPs and explore trends in GPs’ testing behaviour.

**Methods:**

Interactive sessions on HIV and STI using graphical audit and feedback started in 2015. Participating GPs developed improvement plans that were evaluated in follow-up sessions. Laboratory data on STI testing by Amsterdam GPs from 2011 to 2017 were collected for graphical audit and feedback and effect evaluation. The primary outcome was the HIV testing rate: number of HIV tests per 10 000 person-years (PY). Secondary endpoints were chlamydia and gonorrhoea testing rates and HIV positivity ratios.

**Results:**

Since 2015, 41% of GPs participated. HIV testing rate declined from 2011 to 2014 (from 175 to 116 per 10 000 PY), more in women than men (176 to 101 versus 173 to 132), and stabilized from 2015 to 2017. The HIV positivity ratio declined from 0.8% in 2011 to 0.5% in 2017. From 2011 to 2017, chlamydia and gonorrhoea testing rates declined in women (from 618 to 477 per 10 000 PY) but remained stable in men (from 270 to 278).

**Conclusions:**

The stabilization of the downward trend in HIV testing coincided with this educational intervention. Follow-up data are needed to formally assess the intervention’s impact on GP testing behaviour whilst considering contextual factors and secular trends.

**Key Messages**

-  An educational intervention is used to improve HIV testing by general practitioners.
-  The HIV testing rate stabilized after the implementation of the intervention.
-  Follow-up analyses will formally assess long-term impact on HIV testing practices.

## Background

General practitioners (GPs) play a pivotal role in provider-initiated testing and counselling (PITC) for HIV in the Netherlands. About two-thirds of sexually transmitted infection (STI) consultations take place in primary care, and GPs diagnose a third of HIV infections (1). However, opportunities for earlier diagnosis through primary care are often missed. Over 60% of people newly diagnosed with HIV visited their GP in the year prior to diagnosis, and 61% had been diagnosed with indicator conditions (i.e. conditions where HIV testing is recommended) in the 5 years prior to diagnosis (2). This is disappointing, as the STI consultation guideline for GPs, as updated in 2013, includes guidance on appropriate HIV testing strategies (3). Research showed that GPs still perceive barriers towards HIV testing (4–7). Thus, a key approach to eliminating HIV is improving HIV testing strategies by GPs and addressing their barriers to timely testing (7,8).

There were an estimated 23 300 people living with HIV (PLHIV) in the Netherlands in 2018, with 660 new HIV diagnoses, and 47% presenting with a late-stage infection (CD4 <350 cells/mm^3^ or an AIDS-defining event (9)). In Amsterdam, the HIV prevalence is over five times higher than the national prevalence, warranting a city-based approach to curbing the epidemic (10). In 2014, a consortium of stakeholders in HIV prevention and care launched the HIV Transmission Elimination AMsterdam (H-TEAM) initiative (11). The H-TEAM aims to eliminate HIV in Amsterdam through innovative interventions, focussing on all parts of the cascade. Previous studies on educational interventions to improve HIV and STI testing in primary care yielded varying results (12–14). Effects were often of limited size and not sustained. The H-TEAM, therefore, designed a novel educational intervention programme for GPs in Amsterdam using a combination of previously successful and evidence-based elements for educational interventions (15–22). This programme is currently ongoing. The primary research questions in this programme are: ‘what is the uptake and evaluation of the intervention by GPs in Amsterdam? and what is the impact of the intervention on HIV, chlamydia and gonorrhoea testing rates by GPs in Amsterdam?’ Here, we describe the design and implementation of this educational intervention programme, as well as observed trends in HIV and STI testing from 2011 to 2017.

## Methods

### Setting and participants

In 2017, Amsterdam had 844 947 residents and 534 GPs. Over 80% of GPs in Amsterdam work part time. Nearly all residents of the Netherlands are registered with a GP and 78% contact their GP at least once every year (23).

All Amsterdam GPs were invited to participate in the educational intervention. For recruitment, we collaborated with Elaa, which is the key organization for regional primary care support and a well-known and trusted party for GPs. Participating GPs receive accreditation points, which are required for ongoing registration in their specialty.

### Educational intervention

The intervention, in the form of a diagnostic audit meeting (in Dutch: Diagnostisch Toets Overleg; DTO) was designed by a group of GPs and experts on medical education and HIV. Evidence-based elements for effective continuing medical education (CME) to improve physician performance were used in the design, including interactive audit and feedback, multiple educational tools, multiple exposures and small-group sessions (Table 1) (16,21,24).

**Table 1.** Evidence-based elements of successful innovation in health care and their implementation in the H-TEAM’s educational intervention to improve the HIV testing behaviour of GPs in Amsterdam.

Evidence-based elements of effective education	Implementation in the design
Small-scale educational meetings	GPs participate in their local peer group of 5–20 GPs per group.
Implementation process follow-up	DTOs are divided into two consecutive sessions. Implementation of improvement plans made in the first session is evaluated in the second.
Integration of implementation in existing work structures	Improvement plans are made by the GPs for their own practices to ensure effective implementation.
Intrinsic motivational factor focus	GPs choose relevant subjects as a group, receive feedback on their own performance and acquire new competencies through education.
Extrinsic motivational factor focus	GPs are provided with information on differences in testing compared to peers as a form of intercollegiate auditing. GPs participating in the DTOs receive accreditation points for 2 hours.
Target audience involvement	GPs are invited to participate by a well-known and trusted party (Elaa); the sessions are led by a GP from within the group and the expert attending the sessions is from a national GP organization.
Support for the cause	A GP organizes the DTO for their own group of peers to ensure applicability and relevance.
Expert peer trainers	The organizing GP attends a 2-hour teach-the-teacher session to develop a command of the topics discussed.
Interactive sessions	Discussions about differences in testing and barriers and facilitators to implementation are encouraged by the organizing GP; audit and feedback elements are used during the sessions.
Inclusion of multiple teaching strategies	In the sessions, unilateral education by the expert, interactive discussions, quizzes on knowledge, take-home materials and graphical audit and feedback are used to include multiple teaching strategies.

Elaa: key organization for regional primary care support in Amsterdam; DTO: diagnostic audit meeting (in Dutch: Diagnostisch Toets Overleg).

**Figure 1.**
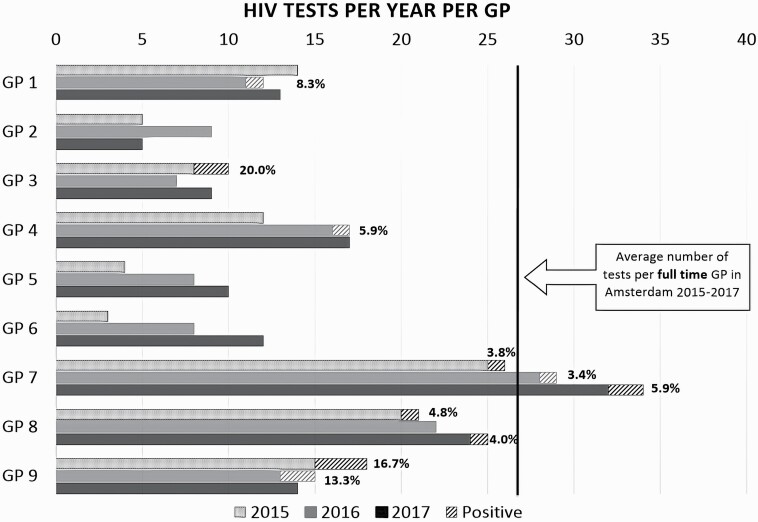
Example of a graphical audit and feedback slide as used in the diagnostic audit meeting to show differences in HIV testing between the participating GPs (adapted from a graphical feedback slide used in the session).

The intervention is organized for existing groups of 5 to 20 GPs and consists of two 2-hour sessions (DTO I and DTO II). During the sessions, trends in HIV and STI prevalence and current guidelines are discussed, as well as appropriate HIV testing strategies, including proactive HIV testing, screening of at-risk groups, indicator condition-based testing and routine HIV testing in new patients. Additionally, practical tips are addressed through interactive discussions, such as strategies to overcome barriers to discussing sexual risk behaviour, and practical tools to facilitate quality STI consultations in the context of busy primary care facilities. Graphical audit and feedback is used to demonstrate differences in testing behaviour between participating GPs relative to the calculated average number of tests by a full-time Amsterdam GP (Fig. 1). The session’s outline and background information are provided in workbooks, which are distributed prior to the session.

Moderation of the sessions is done by GPs that are trained through a specially designed 2-hour teach-the-teacher session to develop extensive knowledge of the topics discussed. A member of the national expert group on HIV and STI in primary care attends to answer in-depth questions. At the end of DTO I, the GPs develop a quality improvement plan for their own practice. In DTO II, approximately 1 year after DTO I depending on the availability of the group, the GPs evaluate its implementation and discuss barriers and facilitators for implementation, further points of improvement and whether HIV and STI testing has improved based on the updated graphical audit and feedback.

### Evaluation of the intervention

After DTO I, the moderating GP completes a questionnaire evaluating the DTO session, including scoring statements on a 10-point scale and open questions, and summarizes the quality improvement plans made. After DTO II, all participating GPs complete a questionnaire evaluating the programme, the self-perceived effect on testing behaviour and adherence to their quality improvement plans. Additional data on the characteristics of these GPs (age, sex, years practising as a GP, type of practice, days working per week and the number of PLHIV in their practice) are collected through this questionnaire.

### Laboratory data

Data on HIV and STI testing by Amsterdam GPs from 2011 onwards are obtained from primary care diagnostic laboratories in Amsterdam. All GPs were given the opportunity to object to the use of their test data for this programme. Data variables include the type of test (HIV, gonorrhoea or chlamydia), anatomical site (blood, urogenital, oral or anorectal), date and outcome of the test, year of birth and sex of the patient and the name and zip code of the ordering GP. Data on tests performed in other contexts, such as antenatal screening, and tests performed by health care professionals other than GPs are excluded. We calculate the HIV, chlamydia and gonorrhoea testing rates per 10 000 person-years (PY; number of tests per 10 000 Amsterdam residents) and the HIV positivity ratios (i.e. the proportion positive among tests performed). To estimate our data coverage, we assess which laboratory participating GPs use for their diagnostics through the evaluative questionnaires. Based on this, we estimate the proportion of Amsterdam GPs that utilize a laboratory that is not included in the data set.

### Analysis

We analysed the initial results of this ongoing intervention and trends in HIV and STI testing by GPs from 2011 to 2017. Scoring statements in the DTO I questionnaires were summarized as means. Open questions were summarized by identified themes. We used a Poisson regression model to examine differences in laboratory testing rates by year with incidence rate ratios (IRRs). Additional analyses stratified by patients’ sex and age categories (<20, 20–34, 35–49, 50–64 and ≥65) were performed. Because the estimated laboratory data coverage was 90–95%, the use of confidence intervals was not deemed appropriate. An effect with a *P*-value of <0.05 was considered statistically significant. Data analysis and effect evaluation were performed using Stata 15 (25).

## Results

### Participating GPs

From February 2015 to October 2019, 30 DTOs were organized, including 22 DTO I and 8 DTO II sessions. In total, 220 GPs attended one or both sessions. This accounts for 41.2% of all GPs in Amsterdam in 2017 (220/534). DTO I was attended by 204 GPs, and 71 attended DTO II. The DTO II sessions are still ongoing. The mean number of participants per session was 9.

### Evaluation of DTO I

Of the 22 groups attending DTO I (including 208 participants), 20 (partially) completed the evaluation (91% of groups; including 181 participants). Overall, the DTO was rated 8.5/10. The content was rated 8.4/10, the method 8.4/10 and the materials 8.6/10. All groups made quality improvement plans for their practice. Six main topics (three on HIV testing and three on STI testing) were identified (Table 2). Five strengths and five points of improvement for the programme were identified (Supplementary table 1).

**Table 2.** Main topics identified in the quality improvement plans made at the end of DTO I.

Quality improvement plans concerning HIV testing	*n*/*N* (%)
- Offer more provider-initiated HIV testing, e.g. by offering an HIV test at consultations concerning other complaints, at the intake procedure, when performing diagnostics for different reasons and through suggesting HIV testing on the TV screens in the waiting room	10/20 (50.0)
- Specifically screen high groups for HIV proactively, including men who have sex with men, people from HIV-endemic countries and patients with positive STI tests in the past	8/20 (40.0)
- Test for HIV more when diagnosing or suspecting an HIV indicator condition, including other STI	7/20 (35.0)
Quality improvement plans concerning STI testing	*n*/*N* (%)
- Be more alert on extragenital STI testing (oral or anal) when indicated	12/20 (60.0)
- Choose type and anatomical location of testing (urogenital, oral, anorectal or blood) based on the guidelines and diagnostic decision tool more	11/20 (55.0)
- Take more detailed sexual histories to more accurately assess risk behaviour to choose the appropriate diagnostics accordingly	9/20 (45.0)

DTO: diagnostic audit meeting (in Dutch: Diagnostisch Toets Overleg).

### HIV and STI testing trends

Seven laboratories provided data on STI testing from 2011 onwards, yielding an estimated 90–95% coverage of tests performed by all Amsterdam GPs. Two GPs opted out; their data were excluded. Data from 2011 to 2017 are reported.

**Figure 2.**
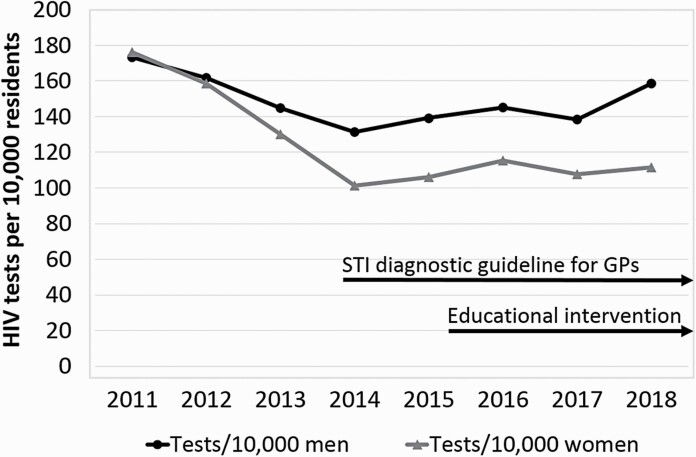
Trends in HIV tests performed by GPs per 10 000 residents of Amsterdam per year by sex and key interventions.

**Figure 3.**
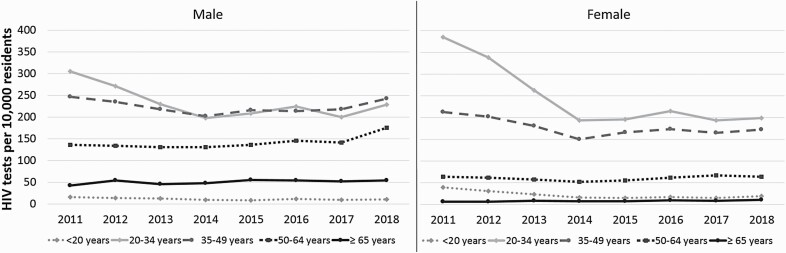
Trends in HIV tests performed by GPs per 10 000 residents of Amsterdam per year by age category and sex.

HIV testing rates declined from 2011 to 2014 (from 174.8 to 116.1 per 10 000 PY, IRR 0.69; Fig. 2). This decline was more pronounced in female patients (176.2 to 101.2 per 10 000 PY, IRR 0.62) than in male patients (173.3 to 131.5 per 10 000 PY, IRR 0.76). HIV testing rates stabilized from 2014 to 2017 (from 116.1 to 122.8 per 10 000 PY, IRR 1.06). The rates were lowest in the extreme age categories (<20 and ≥65). In most age categories (35–49, 50–64 and ≥65), the rates were higher in men than women (Fig. 3). In both sexes, testing in the age categories of 20–34 years and 35–49 years declined from 2011 to 2014. This decline was more pronounced in women than in men.

The overall HIV positivity ratio was low but higher in men than in women. The HIV positivity ratio declined from 2011 to 2017. This decline was more pronounced in men (from 1.2% to 0.7%) than in women (from 0.4% to 0.3%; Supplementary Fig. 1 and Supplementary Table 3).

Chlamydia and gonorrhoea testing rates were higher in women than men. For men, testing rates remained stable for chlamydia (from 269.9 to 286.6 per 10 000 PY, IRR 1.06) and gonorrhoea (from 269.9 to 268.7 per 10 000 PY, IRR 1.00) from 2011 to 2017 (Supplementary Fig. 2 and Supplementary Table 4). For women, they showed a similar pattern as the HIV testing trends, with a decline from 2011 to 2014 (chlamydia: from 615.5 to 471.1, IRR 0.77; gonorrhoea: from 620.8 to 432.7, IRR 0.70) and a partial recovery and stabilization from 2014 to 2017. The recovery was stronger in chlamydia than gonorrhoea, resulting in a difference in testing rates between the two in 2017 (chlamydia: 511.2 per 10 000 PY versus gonorrhoea: 442.9 per 10 000 PY). Although low overall, the testing increased for anorectal chlamydia from 2011 to 2017 (from 4.5 to 16.3 per 10 000 PY, IRR 3.67) and anorectal gonorrhoea (from 4.5 to 15.9 per 10 000 PY, IRR 3.53). This increase was stronger in men than women for both anorectal chlamydia (from 7.1 to 26.6 per 10 000 PY, IRR 3.72, versus from 1.8 to 6.3 per 10 000 PY, IRR 3.42) and anorectal gonorrhoea (from 7.1 to 26.5 per 10 000 PY in men, IRR 3.72, versus from 1.9 to 5.4 per 10 000 PY in women, IRR 2.82; Supplementary Fig. 3 and Supplementary Table 4).

## Discussion

Our educational programme to improve GPs’ HIV testing has been attended by over 41% of Amsterdam GPs since 2015 and has been positively evaluated with an average rating of 8.5/10. Laboratory data showed declining HIV testing by Amsterdam GPs from 2011 to 2014, with a subsequent stabilization from 2014 onwards. The decline was more pronounced in women and in the young and middle age categories (20–34 and 35–49). The HIV positivity ratio declined from 2011 to 2017. There was a decline in chlamydia and gonorrhoea testing rates in women from 2011 to 2014 but, in men, they remained stable. Anorectal chlamydia and gonorrhoea testing rates increased from 2011 to 2017, especially in men.

The initial decline in HIV testing rates coincides with the increase of the Dutch compulsory annual deductible for patients from €170 to €385. GP diagnostics are paid out-of-pocket by patients who have not exhausted their deductible. Meanwhile, HIV testing at STI clinics, which is free for the patient, increased (1). The stabilization of HIV testing from 2014 onwards coincides with the start of our intervention. As such, it might have contributed to this stabilization. Meanwhile, as part of the H-TEAM initiative, GPs received periodic newsletters on appropriate HIV testing from 2014 and contributed to annual free HIV testing weeks from 2015 onwards. Furthermore, the STI consultation guideline for GPs was implemented in September 2013 (3) (Fig. 2). The stabilization in HIV testing also coincides with the implementation of a ceiling on government funding for STI clinics in 2015, leading to constrained access to these clinics. Thus, multiple H-TEAM interventions and contextual factors on the national level could have contributed to the observed trend in HIV testing. As such, no causality of the stabilization in HIV testing can be attributed to this programme. Analyses comparing pre- and post-intervention testing rates and comparing participants and non-participants are needed to determine the effect of this intervention.

The stronger recovery in chlamydia testing rates compared to gonorrhoea testing rates from 2014 onwards is in accordance with the STI consultation guideline for GPs, as it recommends testing only for chlamydia in the absence of STI risk factors (3). In the presence of risk factors, GPs are advised to test for the ‘Big Five’ (chlamydia, gonorrhoea, syphilis, HIV and hepatitis B), with additional testing for STIs like *Trichomonas vaginalis*, lymphogranuloma venereum, *Mycoplasma genitalium* and hepatitis C, if indicated. The guideline also emphasizes the importance of a detailed sexual history to identify the indications for extragenital STI testing. Accordingly, our data reveal increased anorectal chlamydia and gonorrhoea testing, predominantly in men, although these rates remain low.

Comparing HIV testing by Amsterdam GPs to other high-prevalence areas in Europe reveals mixed results. In Haringey, London, an area with a similar HIV prevalence as Amsterdam (7.5 versus 7.3 per 1000 residents, respectively (9,26)), an intervention amongst GPs yielded a testing rate of 59.9 per 10 000 PY in 2012 (17). That year, the testing rate by Amsterdam GPs was over 2.5 times higher. Conversely, the HIV testing rate by GPs in France in 2013 was 580.7 per 10 000 PY in residents aged 15–70 years (27), while the testing rate by Amsterdam GPs was 137.3 per 10 000 PY for all ages. This difference is possibly due to diagnostics being free in France, while, in the Netherlands, a deductible is compulsory.

Our ongoing programme has several strengths. The intervention uses multiple evidence-based elements for performance improvement (Table 1): graphical audit and feedback is a proven effective intervention (28); small-scale interactive sessions are more successful than traditional strategies, such as one-time lectures; and multifaceted programmes are recommended, including elements such as group discussion and designing quality improvement plans (15–21). We aimed to create a sustainable effect by adopting those elements that were proven sustainably effective in comparable settings (29). Additionally, a GP moderating the DTO for its peers elicits a more open discussion, allowing for more effective adherence to the quality improvement plans (30). During the sessions, we address other STIs aside from HIV to make participation more rewarding and to improve GPs’ STI consultations as a whole. Dutch GPs experience heavy workloads and have limited time for professional development (31), so the high participation ratio so far indicates that GPs are interested in these topics. This high participation ratio could additionally be explained by our collaboration with the regional primary care support organization Elaa for the recruitment of participants as they are a well-known and trusted party for GPs. As this project is ongoing, the number of participants to DTO II is still rising. For this project, a unique data set was assembled, providing novel and valuable insight into the role of primary care in HIV testing. Data on HIV and STI testing were not readily available for primary care, and only data on HIV and STI diagnoses made in primary care are provided through national surveillance. Retrieving data from laboratories yielded an estimated 90–95% of STI tests ordered by Amsterdam GPs since 2011. Finally, as these data contain actual test data, there is no risk of recall bias (as in interviews) or registration bias (as for electronic health records).

Our study is limited by the fact that there is considerable variation in practice size between GPs (e.g. GPs working part time) and over time (e.g. maternity leave). Consequently, differences in testing behaviour by GPs in the graphical feedback shown during the sessions warrant cautious interpretation. This is reflected in the DTO I evaluations, where 35% of participants named the skewed or incomplete graphical feedback as a point of improvement. We additionally present the calculated mean number of tests per full-time GP so that GPs can compare their testing behaviour to this mean considering their own practice size. Additionally, although we aim to improve PITC by GPs, we cannot distinguish between provider-initiated tests and patient-requested tests (32). It is possible that changes in HIV testing are due to changes in patients requesting HIV testing as opposed to changes in GPs proactively testing for HIV. Furthermore, the changes in testing rates could not be corrected for patients’ risk factors for HIV acquisition (e.g. ethnicity or sexual behaviour). Consequently, a declining testing rate is not incompatible with a more targeted testing strategy by GPs. Data on these risk factors could help more accurately assess whether testing behaviour is improving, but collection of these data is limited by European privacy regulations. However, the declining HIV incidence in Amsterdam is reflected in the declining HIV positivity ratio in our data. This suggests that GPs testing behaviour is not becoming more targeted as we would then find a more stable positivity ratio. Although the number of undiagnosed PLHIV in the Netherlands is decreasing, 47% of new HIV diagnoses are late-stage infections (9). Thus, improving GPs’ testing strategies to ensure timely testing of patients at risk, presenting with symptoms of acute HIV or with an indicator condition, remains of utmost importance. Finally, we did not include electronic prompts as a strategy to increase HIV testing. During the design of this intervention, GPs were resistant to such an intervention due to ‘prompt fatigue’. Although current evidence on this approach shows variable results (33–35), this strategy might further improve GPs’ testing practices in the future.

## Conclusions

We described the rationale, design and implementation of an evidence-based educational intervention to improve provider-initiated HIV testing by GPs in Amsterdam. A stabilization in HIV testing coincided with the start of our intervention, although no inference on causality can be made. Analyses of follow-up data from this ongoing project, including qualitative and quantitative data, will determine the intervention’s effectiveness to improve GPs’ testing behaviour, whilst considering contextual factors and secular trends. Facilitating earlier diagnosis and treatment of HIV in primary care will ultimately help achieve our ambitious goal of eliminating HIV in Amsterdam, the Netherlands.

## Supplementary Material

cmaa139_suppl_Supplementary-Material

## Data Availability

Data will be shared on request to the corresponding author.

## Declaration

Funding: PROTest 2.0 is funded by Aidsfonds (grant number: P- 42702) and the H-TEAM. The H-TEAM initiative is supported by Aidsfonds (grant number: 2013169), Stichting AmsterdamDiner Foundation, Bristol-Myers Squibb International Corp. (study number: AI424-541), Gilead Sciences Europe Ltd (grant number: PA-HIV-PREP-16-0024), Gilead Sciences (protocol numbers: CO-NL-276-4222, CO-US-276-1712), Janssen Pharmaceutica (reference number: PHNL/JAN/0714/0005b/1912fde), M.A.C AIDS Fund, ViiV Healthcare (PO numbers: 3000268822, 3000747780) and ZonMw (grant number: 522002003).

Ethics approval: the Medical Ethics Committee of the Amsterdam UMC determined that this study does not meet the definition of medical research involving human subjects under Dutch law. All GPs were given opportunity to object to the use of their data. Participating GPs provided informed consent for the use of their evaluations for research purposes.
