# Correction to: Rationale, design and initial results of an educational intervention to improve provider-initiated HIV testing in primary care

**DOI:** 10.1093/fampra/cmad007

**Published:** 2023-01-28

This is a correction notice only for: Rationale, design and initial results of an educational intervention to improve provider-initiated HIV testing in primary care, *Family Practice*, Volume 38, Issue 4, August 2021, Pages 441–447, https://doi.org/10.1093/fampra/cmaa139

In the originally published version of this manuscript, there was an error in data from 2018 being presented in Figures 2 and 3. This should be only up to (and including) 2017.

Figure 2 should read:


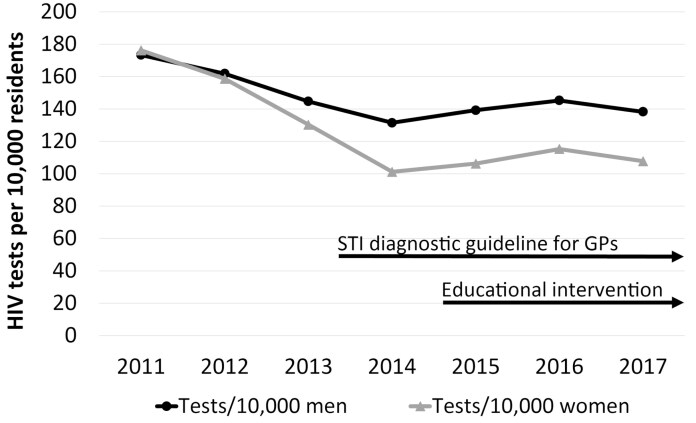


instead of: 


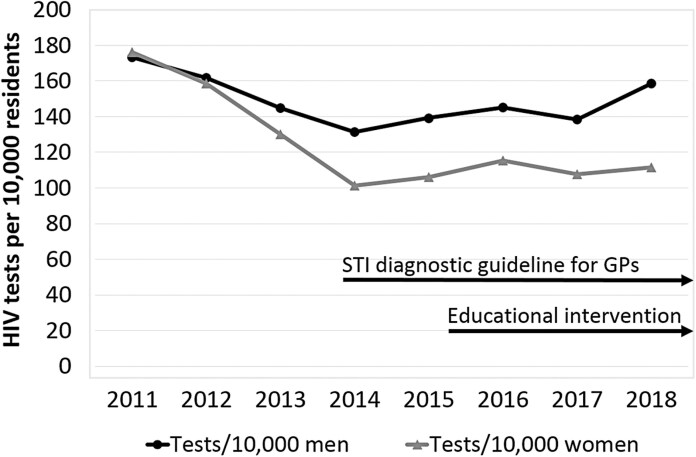


Figure 3 should read:


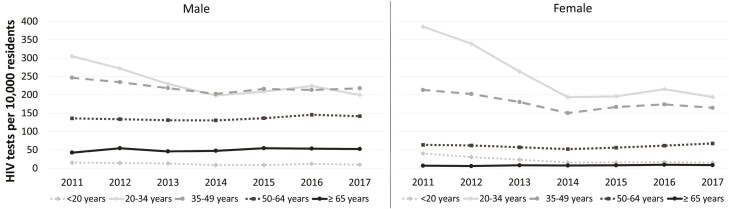


instead of:


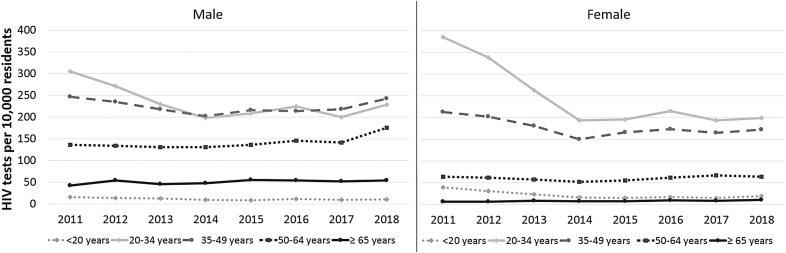


In section **HIV and STI testing trends**, second paragraph, second sentence, a reference link to supplementary material was missing. This should read: 

“This decline was more pronounced in female patients (176.2 to 101.2 per 10 000 PY, IRR 0.62) than in male patients (173.3 to 131.5 per 10 000 PY, IRR 0.76, supplementary table 2).”

instead of:

“This decline was more pronounced in female patients (176.2 to 101.2 per 10 000 PY, IRR 0.62) than in male patients (173.3 to 131.5 per 10 000 PY, IRR 0.76).”

The **Supplementary data** section had errors both in the data included and labelling of tables. The file is now replaced with:

The errors were: The data for Supplementary table 1 was missing. The first dataset of Supplementary table 2 was mislabelled as Table 1. The second dataset of Supplementary table 2 and Supplementary table 3’s dataset were linked and together mislabelled as Table 2. Supplementary table 4 was mislabelled as Table 3. Table 4 (*Positivity ratio of all chlamydia & gonorrhoea testing by Amsterdam GPs per year by sex*) was published in error and is now deleted from the corrected Supplemental data file.

These details have been corrected only in this correction notice to preserve the published version of record.

## Supplementary Material

cmad007_suppl_Supplementary_Material

