# Supplementary material for: Rationale, design and initial results of an educational intervention to improve provider-initiated HIV testing in primary care
Source: Fam Pract. 2020 Dec 26;38(4):441–7. doi: 10.1093/fampra/cmaa139 (PMC8317217; doi:10.1093/fampra/cmaa139)
Supplement: cmaa139_suppl_Supplementary-Material [file cmaa139_suppl_supplementary-material.docx]

### **SUPPLEMENT TO:**

**RATIONALE, DESIGN AND INITIAL RESULTS OF THE H-TEAM’S EDUCATIONAL INTERVENTION TO INCREASE PROVIDER-INITIATED HIV TESTING IN PRIMARY CARE IN AMSTERDAM, THE NETHERLANDS**

**Authors:**

S.J. Bogers^1^, M.F. Schim van der Loeff^1,2^, N. van Dijk^3^, K. Groen^4^, M.L. Groot Bruinderink^2,4^, G.J. de Bree^1,4^, P. Reiss^1,4,5,6^, S.E. Geerlings^1^ and J.E.A.M van Bergen^3,7^ on behalf of the PROTest 2.0 project in the HIV Transmission Elimination AMsterdam (H-TEAM) Consortium

**Affiliations:**

^1^ Department of Internal Medicine, Division of Infectious Diseases, Amsterdam University Medical Centers, location Academic Medical Center, University of Amsterdam, the Netherlands.

^2^ Department of Infectious Diseases, Public Health Service of Amsterdam, Amsterdam, the Netherlands.

^3^ Department of General Practice, Amsterdam University Medical Centers, location Academic Medical Center, University of Amsterdam, the Netherlands.

^4^ Amsterdam Institute for Global Health and Development, Amsterdam, the Netherlands.

^5^ Department of Global Health, Amsterdam University Medical Centers, location Academic Medical Center, University of Amsterdam, the Netherlands.

^6^ HIV Monitoring Foundation, Amsterdam, the Netherlands.

^7^ STI AIDS Netherlands, Amsterdam, the Netherlands.

**Content:**

Figures: 3.

Tables: 4.

**Figure 1:** Trends in positivity ratio of HIV tests performed by general practitioners by sex (percentage of positive HIV tests amongst all HIV tests performed)

**Figure 2:** Trends in chlamydia & gonorrhoea tests performed by GPs per 10,000 person-years by sex. CT = *chlamydia trachomatis*, NG = *Neisseria gonorrhoeae*

**Figure 3:** Trends in anorectal chlamydia & gonorrhoea tests performed by GPs per 10,000 person-years by sex. ACT = anorectal *chlamydia trachomatis*, ANG = anorectal *Neisseria gonorrhoeae*

**Table 1: HIV testing rates by GPs per 10,000 person-years** **by age category and sex**

| **Female** | | | | | | |
| --- | --- | --- | --- | --- | --- | --- |
|  | **<20 years** | **20-34 years** | **35-49 years** | **50-64 years** | **≥ 65 years** | **Total female** |
| **2011** | 39.77 | 385.58 | 213.08 | 63.27 | 6.51 | 176.21 |
| **2012** | 30.26 | 338.81 | 201.80 | 61.64 | 5.74 | 158.59 |
| **2013** | 22.89 | 263.01 | 180.43 | 57.04 | 7.94 | 130.22 |
| **2014** | 15.36 | 193.28 | 149.99 | 51.78 | 7.40 | 101.16 |
| **2015** | 15.28 | 195.76 | 166.42 | 55.59 | 7.65 | 106.20 |
| **2016** | 16.48 | 215.42 | 173.91 | 61.29 | 9.16 | 115.26 |
| **2017** | 14.78 | 193.77 | 164.56 | 67.23 | 8.45 | 107.68 |

| **Male** | | | | | | |
| --- | --- | --- | --- | --- | --- | --- |
|  | **<20 years** | **20-34 years** | **35-49 years** | **50-64 years** | **≥ 65 years** | **Total male** |
| **2011** | 15.49 | 305.20 | 246.40 | 135.95 | 42.55 | 173.29 |
| **2012** | 14.07 | 271.61 | 234.78 | 133.42 | 54.59 | 161.80 |
| **2013** | 12.89 | 229.65 | 218.21 | 131.15 | 45.89 | 144.69 |
| **2014** | 8.89 | 198.19 | 202.55 | 130.31 | 47.43 | 131.46 |
| **2015** | 8.73 | 208.93 | 215.77 | 136.41 | 54.66 | 139.20 |
| **2016** | 12.01 | 224.17 | 213.52 | 145.80 | 53.59 | 145.24 |
| **2017** | 9.60 | 199.49 | 217.86 | 141.75 | 52.23 | 138.26 |

**Table 2: Positivity ratio** **of all HIV tests ordered by Amsterdam GPs per year by sex**

|  | **Male % positive** | **Female % positive** | **Total % positive** |
| --- | --- | --- | --- |
| **2011** | 1.22 | 0.39 | 0.79 |
| **2012** | 0.92 | 0.42 | 0.67 |
| **2013** | 1.04 | 0.40 | 0.73 |
| **2014** | 1.12 | 0.22 | 0.72 |
| **2015** | 0.92 | 0.36 | 0.68 |
| **2016** | 0.90 | 0.25 | 0.61 |
| **2017** | 0.71 | 0.26 | 0.51 |

**Table 3: Chlamydia and gonorrhoea testing rates by GPs per 10,000 person-years** **by sex**

|  | **Chlamydia** | | | **Gonorrhoea** | | |
| --- | --- | --- | --- | --- | --- | --- |
|  | **Male** | **Female** | **Total** | **Male** | **Female** | **Total** |
| **2011** | 269.85 | 615.50 | 445.38 | 269.93 | 620.80 | 448.11 |
| **2012** | 272.90 | 613.28 | 445.70 | 271.80 | 614.63 | 445.84 |
| **2013** | 260.82 | 538.43 | 401.84 | 256.67 | 525.35 | 393.16 |
| **2014** | 248.86 | 471.12 | 361.64 | 239.19 | 432.72 | 337.40 |
| **2015** | 269.68 | 486.55 | 379.70 | 254.84 | 457.72 | 357.76 |
| **2016** | 288.99 | 525.15 | 408.54 | 269.07 | 473.92 | 372.77 |
| **2017** | 286.61 | 511.20 | 400.06 | 268.72 | 442.90 | 356.71 |
|  | **Anorectal** **chlamydia** | | | **Anorectal** **gonorrhoea** | | |
|  | **Male** | **Female** | **Total** | **Male** | **Female** | **Total** |
| **2011** | 7.14 | 1.84 | 4.45 | 7.14 | 1.92 | 4.49 |
| **2012** | 8.30 | 1.57 | 4.89 | 8.38 | 1.65 | 4.96 |
| **2013** | 8.42 | 2.00 | 5.16 | 8.34 | 2.04 | 5.14 |
| **2014** | 11.62 | 2.21 | 6.84 | 11.54 | 2.07 | 6.73 |
| **2015** | 15.88 | 4.22 | 9.97 | 15.96 | 4.03 | 9.91 |
| **2016** | 21.21 | 6.47 | 13.75 | 21.31 | 5.62 | 13.36 |
| **2017** | 26.55 | 6.30 | 16.32 | 26.52 | 5.41 | 15.86 |

**Table 4: Positivity ratio** **of all chlamydia and gonorrhoea testing by Amsterdam GPs per year by sex**

|  | **Chlamydia** | | | **Gonorrhoea** | | |
| --- | --- | --- | --- | --- | --- | --- |
|  | **Male**  **% positive** | **Female**  **% positive** | **Total**  **% positive** | **Male**  **% positive** | **Female**  **% positive** | **Total**  **% positive** |
| **2011** | 7.57 | 5.13 | 5.86 | 3.19 | 0.90 | 1.58 |
| **2012** | 8.23 | 5.41 | 6.26 | 2.98 | 1.07 | 1.65 |
| **2013** | 8.58 | 5.28 | 6.34 | 2.65 | 0.77 | 1.37 |
| **2014** | 9.19 | 5.71 | 6.89 | 3.15 | 0.63 | 1.51 |
| **2015** | 10.02 | 6.75 | 7.89 | 3.67 | 0.76 | 1.78 |
| **2016** | 9.68 | 6.83 | 7.83 | 3.75 | 0.75 | 1.82 |
| **2017** | 9.28 | 7.09 | 7.86 | 3.54 | 0.87 | 1.86 |
|  | **Anorectal chlamydia** | | | **Anorectal gonorrhoea** | | |
|  | **Male**  **% positive** | **Female**  **% positive** | **Total**  **% positive** | **Male**  **% positive** | **Female**  **% positive** | **Total**  **% positive** |
| **2011** | 9.85 | 6.85 | 9.22 | 9.85 | 2.63 | 8.29 |
| **2012** | 13.31 | 6.35 | 12.18 | 10.74 | 3.03 | 9.44 |
| **2013** | 12.39 | 4.94 | 10.92 | 10.37 | 1.20 | 8.52 |
| **2014** | 12.28 | 4.40 | 10.99 | 8.03 | 1.18 | 6.96 |
| **2015** | 11.20 | 10.23 | 10.99 | 9.75 | 1.79 | 8.11 |
| **2016** | 9.62 | 12.09 | 10.21 | 13.80 | 1.69 | 11.22 |
| **2017** | 10.27 | 13.01 | 10.80 | 9.92 | 1.73 | 8.51 |
