# Supplementary material for: Correction to: Rationale, design and initial results of an educational intervention to improve provider-initiated HIV testing in primary care
Source: Fam Pract. 2023 Jan 28;42(3):cmad007. doi: 10.1093/fampra/cmad007 (PMC12014900; doi:10.1093/fampra/cmad007)
Supplement: cmad007_suppl_Supplementary_Material [file cmad007_suppl_supplementary_material.docx]

### **SUPPLEMENT TO:**

**RATIONALE, DESIGN AND INITIAL RESULTS OF THE H-TEAM’S EDUCATIONAL INTERVENTION TO INCREASE PROVIDER-INITIATED HIV TESTING IN PRIMARY CARE IN AMSTERDAM, THE NETHERLANDS**

**Authors:**

S.J. Bogers^1^, M.F. Schim van der Loeff^1,2^, N. van Dijk^3^, K. Groen^4^, M.L. Groot Bruinderink^2,4^, G.J. de Bree^1,4^, P. Reiss^1,4,5,6^, S.E. Geerlings^1^ and J.E.A.M van Bergen^3,7^ on behalf of the PROTest 2.0 project in the HIV Transmission Elimination AMsterdam (H-TEAM) Consortium

**Affiliations:**

^1^ Department of Internal Medicine, Division of Infectious Diseases, Amsterdam University Medical Centers, location Academic Medical Center, University of Amsterdam, the Netherlands.

^2^ Department of Infectious Diseases, Public Health Service of Amsterdam, Amsterdam, the Netherlands.

^3^ Department of General Practice, Amsterdam University Medical Centers, location Academic Medical Center, University of Amsterdam, the Netherlands.

^4^ Amsterdam Institute for Global Health and Development, Amsterdam, the Netherlands.

^5^ Department of Global Health, Amsterdam University Medical Centers, location Academic Medical Center, University of Amsterdam, the Netherlands.

^6^ HIV Monitoring Foundation, Amsterdam, the Netherlands.

^7^ STI AIDS Netherlands, Amsterdam, the Netherlands.

**Content:**

Figures: 3.

Tables: 4.

**Suppl Figure 1:** Trends in positivity ratio of HIV tests performed by general practitioners by sex (percentage of positive HIV tests amongst all HIV tests performed)

**
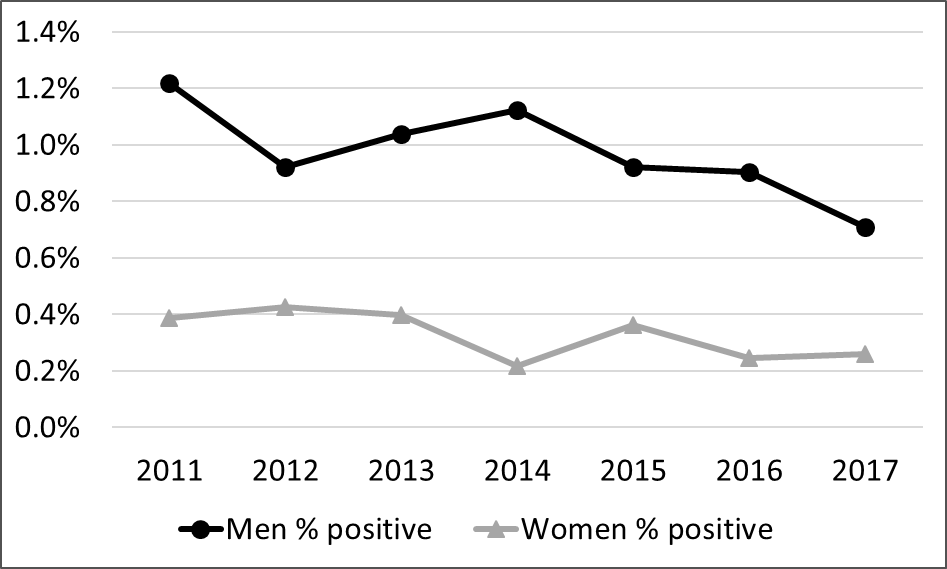
**

**Suppl. Figure 2:** Trends in chlamydia & gonorrhoea tests performed by GPs per 10,000 person-years by sex. CT = *chlamydia trachomatis*, NG = *Neisseria gonorrhoeae*


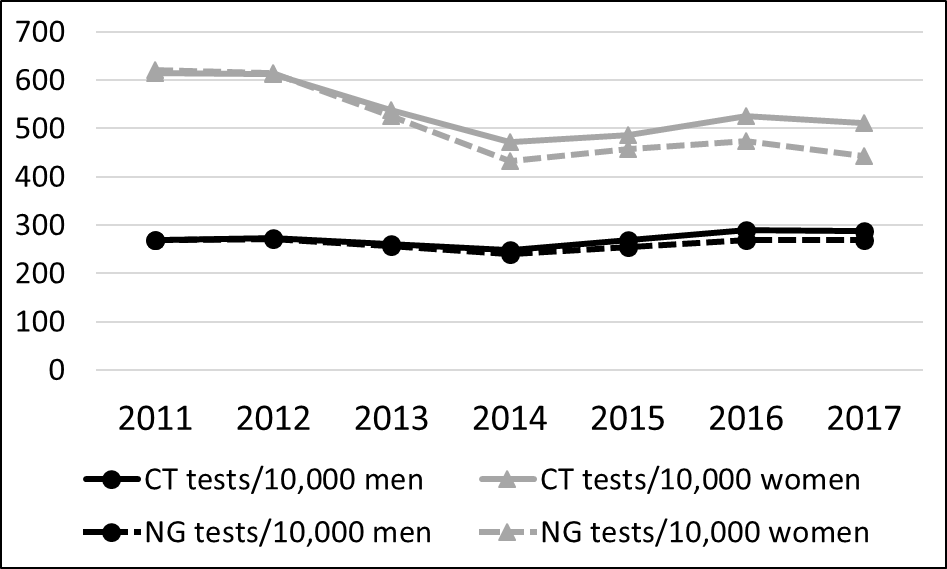


**Suppl. Figure 3:** Trends in anorectal chlamydia & gonorrhoea tests performed by GPs per 10,000 person-years by sex. ACT = anorectal *chlamydia trachomatis*, ANG = anorectal *Neisseria gonorrhoeae*


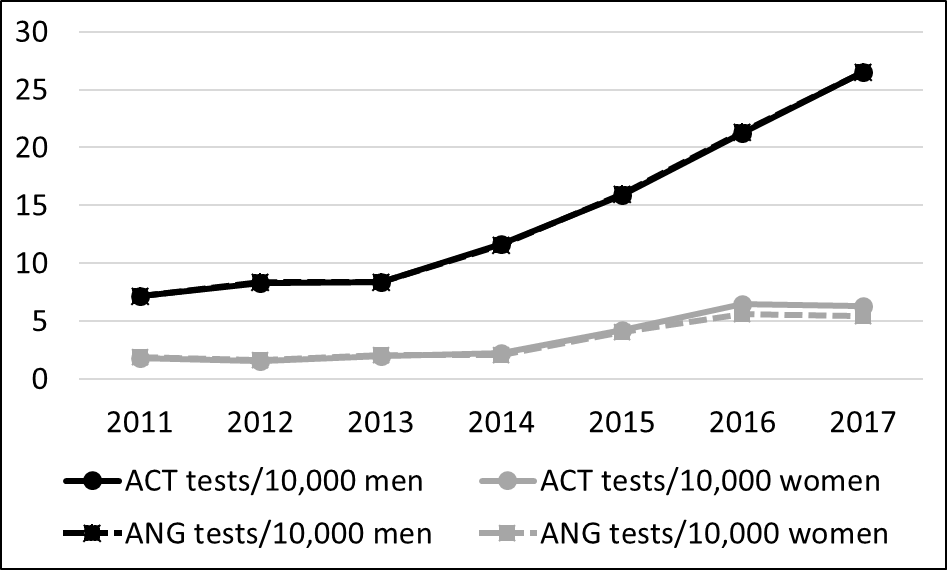


**Suppl. Table 1:** Strengths and points of improvement for the DTO I sessions mentioned in evaluation by 20 participating groups

| **Strengths of the DTO sessions** | **n (%)** |
| --- | --- |
| Gaining more knowledge and awareness on the several topics | 8 (40) |
| Interactive design of the session | 4 (20) |
| Discussing the diagnostic decision tool for STI testing | 3 (15) |
| Graphical audit and feedback and discussion | 2 (10) |
| Group discussion on stigma and barriers to discussing STI with the patient | 2 (10) |
| **Points of improvement for the DTO sessions** | **n (%)** |
| The graphical feedback showed incomplete or skewed data | 7 (35) |
| Participants wanted more information on epidemiology of STI in their area | 3 (15) |
| Participants wanted more exemplary practice cases | 2 (10) |
| Participants wanted a shorter session | 2 (10) |
| Participants wanted a longer session | 2 (10) |

DTO = diagnostic audit meeting (in Dutch: Diagnostisch Toets Overleg). STI = sexually transmitted infection.

**Suppl. Table 2:** HIV testing rates by GPs per 10,000 person-years by age category and sex

| **Male** | | | | | | |
| --- | --- | --- | --- | --- | --- | --- |
|  | **<20 years** | **20-34 years** | **35-49 years** | **50-64 years** | **≥ 65 years** | **Total male** |
| **2011** | 15.49 | 305.20 | 246.40 | 135.95 | 42.55 | 173.29 |
| **2012** | 14.07 | 271.61 | 234.78 | 133.42 | 54.59 | 161.80 |
| **2013** | 12.89 | 229.65 | 218.21 | 131.15 | 45.89 | 144.69 |
| **2014** | 8.89 | 198.19 | 202.55 | 130.31 | 47.43 | 131.46 |
| **2015** | 8.73 | 208.93 | 215.77 | 136.41 | 54.66 | 139.20 |
| **2016** | 12.01 | 224.17 | 213.52 | 145.80 | 53.59 | 145.24 |
| **2017** | 9.60 | 199.49 | 217.86 | 141.75 | 52.23 | 138.26 |
| **Female** | | | | | | |
|  | **<20 years** | **20-34 years** | **35-49 years** | **50-64 years** | **≥ 65 years** | **Total female** |
| **2011** | 39.77 | 385.58 | 213.08 | 63.27 | 6.51 | 176.21 |
| **2012** | 30.26 | 338.81 | 201.80 | 61.64 | 5.74 | 158.59 |
| **2013** | 22.89 | 263.01 | 180.43 | 57.04 | 7.94 | 130.22 |
| **2014** | 15.36 | 193.28 | 149.99 | 51.78 | 7.40 | 101.16 |
| **2015** | 15.28 | 195.76 | 166.42 | 55.59 | 7.65 | 106.20 |
| **2016** | 16.48 | 215.42 | 173.91 | 61.29 | 9.16 | 115.26 |
| **2017** | 14.78 | 193.77 | 164.56 | 67.23 | 8.45 | 107.68 |

**Suppl. Table 3:** Positivity ratio of all HIV tests ordered by Amsterdam GPs per year by sex

|  | **Male % positive** | **Female % positive** | **Total % positive** |
| --- | --- | --- | --- |
| **2011** | 1.22 | 0.39 | 0.79 |
| **2012** | 0.92 | 0.42 | 0.67 |
| **2013** | 1.04 | 0.40 | 0.73 |
| **2014** | 1.12 | 0.22 | 0.72 |
| **2015** | 0.92 | 0.36 | 0.68 |
| **2016** | 0.90 | 0.25 | 0.61 |
| **2017** | 0.71 | 0.26 | 0.51 |

**Suppl. Table 4:** Chlamydia and gonorrhoea testing rates by GPs per 10,000 person-years by sex

|  | **Chlamydia** | | | **Gonorrhoea** | | |
| --- | --- | --- | --- | --- | --- | --- |
|  | **Male** | **Female** | **Total** | **Male** | **Female** | **Total** |
| **2011** | 269.85 | 615.50 | 445.38 | 269.93 | 620.80 | 448.11 |
| **2012** | 272.90 | 613.28 | 445.70 | 271.80 | 614.63 | 445.84 |
| **2013** | 260.82 | 538.43 | 401.84 | 256.67 | 525.35 | 393.16 |
| **2014** | 248.86 | 471.12 | 361.64 | 239.19 | 432.72 | 337.40 |
| **2015** | 269.68 | 486.55 | 379.70 | 254.84 | 457.72 | 357.76 |
| **2016** | 288.99 | 525.15 | 408.54 | 269.07 | 473.92 | 372.77 |
| **2017** | 286.61 | 511.20 | 400.06 | 268.72 | 442.90 | 356.71 |
|  | **Anorectal** **chlamydia** | | | **Anorectal** **gonorrhoea** | | |
|  | **Male** | **Female** | **Total** | **Male** | **Female** | **Total** |
| **2011** | 7.14 | 1.84 | 4.45 | 7.14 | 1.92 | 4.49 |
| **2012** | 8.30 | 1.57 | 4.89 | 8.38 | 1.65 | 4.96 |
| **2013** | 8.42 | 2.00 | 5.16 | 8.34 | 2.04 | 5.14 |
| **2014** | 11.62 | 2.21 | 6.84 | 11.54 | 2.07 | 6.73 |
| **2015** | 15.88 | 4.22 | 9.97 | 15.96 | 4.03 | 9.91 |
| **2016** | 21.21 | 6.47 | 13.75 | 21.31 | 5.62 | 13.36 |
| **2017** | 26.55 | 6.30 | 16.32 | 26.52 | 5.41 | 15.86 |
